# Post weaning diarrhea in pigs: risk factors and non-colistin-based control strategies

**DOI:** 10.1186/s13028-017-0299-7

**Published:** 2017-05-19

**Authors:** Mohamed Rhouma, John Morris Fairbrother, Francis Beaudry, Ann Letellier

**Affiliations:** 1 Department of Pathology and Microbiology, Faculty of Veterinary Medicine, University of Montreal, 3200 Sicotte Street, Saint-Hyacinthe, QC J2S 2M2 Canada; 2 Department of Veterinary Biomedicine, Faculty of Veterinary Medicine, University of Montreal, 3200 Sicotte Street, Saint-Hyacinthe, QC J2S 2M2 Canada

**Keywords:** Post-weaning diarrhea, Pigs, *E. coli*, Colistin, Resistance, Alternatives

## Abstract

Post-weaning diarrhea (PWD) is one of the most serious threats for the swine industry worldwide. It is commonly associated with the proliferation of enterotoxigenic *Escherichia coli* in the pig intestine. Colistin, a cationic antibiotic, is widely used in swine for the oral treatment of intestinal infections caused by *E. coli*, and particularly of PWD. However, despite the effectiveness of this antibiotic in the treatment of PWD, several studies have reported high rates of colistin resistant *E. coli* in swine. Furthermore, this antibiotic is considered of very high importance in humans, being used for the treatment of infections due to multidrug-resistant (MDR) Gram-negative bacteria (GNB). Moreover, the recent discovery of the *mcr*-*1* gene encoding for colistin resistance in *Enterobacteriaceae* on a conjugative stable plasmid has raised great concern about the possible loss of colistin effectiveness for the treatment of MDR-GNB in humans. Consequently, it has been proposed that the use of colistin in animal production should be considered as a last resort treatment only. Thus, to overcome the economic losses, which would result from the restriction of use of colistin, especially for prophylactic purposes in PWD control, we believe that an understanding of the factors contributing to the development of this disease and the putting in place of practical alternative strategies for the control of PWD in swine is crucial. Such alternatives should improve animal gut health and reduce economic losses in pigs without promoting bacterial resistance. The present review begins with an overview of risk factors of PWD and an update of colistin use in PWD control worldwide in terms of quantities and microbiological outcomes. Subsequently, alternative strategies to the use of colistin for the control of this disease are described and discussed. Finally, a practical approach for the control of PWD in its various phases is proposed.

## Background

Post-weaning diarrhea (PWD) due to *Escherichia coli* is an economically important disease in pig production worldwide, affecting pigs during the first 2 weeks after weaning and characterized by sudden death or diarrhea, dehydration, and growth retardation in surviving piglets [1, 2]. Furthermore, many stress factors associated with the weaning period, such as removal from the sow, dietary changes, adapting to a new environment, mixing of pigs from different farms and histological changes in the small intestine, may negatively affect the response of immune system and lead to an intestinal gut dysfunction in pigs [3–5]. Post-weaning diarrhea is usually associated with proliferation of enterotoxigenic *E. coli* (ETEC) [2, 6]. This pathotype is characterized by the production of enterotoxins and adhesins, both essential for disease development [7], the predominant adhesins in PWD being F4 and F18 [6, 8]. Small intestinal adhesion and subsequent colonization by ETEC in pigs is mediated by F4 or F18 specific receptors, the existence and function of these receptors being crucial to determine the susceptibility of pigs to ETEC infections [7]. The predominant serogroup of ETEC associated with PWD in pigs worldwide is O149, commonly in the combination O149: LT: STa: STb: EAST1: F4ac [2]. Colistin, a polymyxin antibiotic produced by *Paenibacillus polymyxa* var *colistinus* [9], is widely used for the control of PWD in pigs [10]. However, in humans this antibiotic is now considered as the last therapeutic option for the treatment of infections caused by multidrug-resistant Gram-negative bacteria (MDR-GNB) such as *Pseudomonas aeruginosa*, *Acinetobacter baumannii*, *Klebsiella pneumoniae* and *Enterobacter* species [11, 12].

On the other hand, in the last several years, studies have reported the isolation of colistin-resistant *E. coli* from pigs [13, 14], the proportion reaching 35% in some countries [15]. Until recently, resistance to colistin had only been associated with non-transferable genome mediated mutation. However, in 2015, a stable plasmid-mediated gene, *mcr*-*1,* encoding a phosphoethanolamine transferase conferring resistance to colistin was identified in certain GNB, such as *E. coli* and *Salmonella*, isolated from various origins including farm animals, raw meat and humans, in several countries [16–18]. The discovery of a mechanism for horizontal transfer of colistin resistance, and hence the potential for interspecies transfers, gave rise to a strong reaction in the scientific community regarding the potential reduction of colistin effectiveness in human medicine [19]. Food producing animals, and in particular pigs, have been singled out as the most potential reservoirs for spread and amplification of colistin resistance [19]. Thus, scientists and regulatory agencies such as the European Medicine Agency (EMA) have recommended reducing the use of colistin in animal production and to restrict its use to the treatment of sick animals as a last resort option [20]. In addition, several studies have reported coexistence of the *mcr*-*1* gene with genes encoding the production of extended-spectrum β-lactamase (ESBL) and carbapenemase enzymes [21–23]. This constitutes an additional degree of concern about the risk of spread of resistance against antimicrobials of very high importance in human medicine. Furthermore, a high prevalence of ESBL-positive *E. coli* isolated from PWD piglets has been reported [24]. Taken together, these findings underline the need to better understand PWD risk factors and to find alternatives to antimicrobials and particularly to colistin in pigs for the control of PWD in order to manage antimicrobial resistance and maintain at the same time livestock productivity. Hence, the aim of the present review was to provide an overview of risk factors of PWD as well as an update of information on the extent of colistin use in PWD control worldwide in terms of quantities and microbiological outcomes. In addition, alternative strategies to the use of colistin for the control of this disease are described and discussed. Finally, a practical approach is proposed for the control of the PWD in its various phases.

The prevalence of colistin resistance in pigs and the possible link between colistin pharmacokinetic/pharmacodynamic (PK/PD) and emergence of resistance in *Enterobacteriaceae* in swine, as well as the aspects that should be considered to ensure judicious use of colistin in swine production, have been investigated in our last two reviews [18, 25].

### Search strategy and selection criteria

Articles published in peer-reviewed journals were searched in the international online databases PubMed, Web of Science, and Scopus. The studies were selected based on language (English or French) and accessibility to the full manuscript version. Literature was retrieved through an electronic search, starting from 1980 to the present. Relevant scientific papers were identified using the keyword combinations (piglet OR swine OR pig OR weaned OR sows AND (post-weaning diarrhea), (post-weaning), (*E. coli*), (colistin), (colistin resistance), (colistin use), (colistin indications), AND (pig OR swine OR weaned pigs OR antibiotics in pigs OR colistin in pigs OR *E. coli* in pigs OR post-weaning diarrhea OR weanling diet in pigs AND (feed strategies) OR (alternatives measures) OR (alternatives to antibiotics) OR (preventive strategies) OR (additives). All searches were performed from September to November 2016. In total, 389 nonduplicate articles were found. After applying the inclusion and exclusion criteria, 271 citations were considered potentially eligible for inclusion in this review.

### Risk factors for post-weaning diarrhea in pigs

Post-weaning diarrhea is an economically important enteric disease in pigs due to financial losses [1]. This disease occurs most frequently within the 2 weeks after weaning and is characterized by a profuse diarrhea, dehydration, significant mortality and loss of body weight of surviving pigs [2]. Mortality associated with this disease may reach 20–30% over a 1- to 2-month time span among infected weaned pigs during acute outbreaks of PWD [1].

**Fig. 1:**
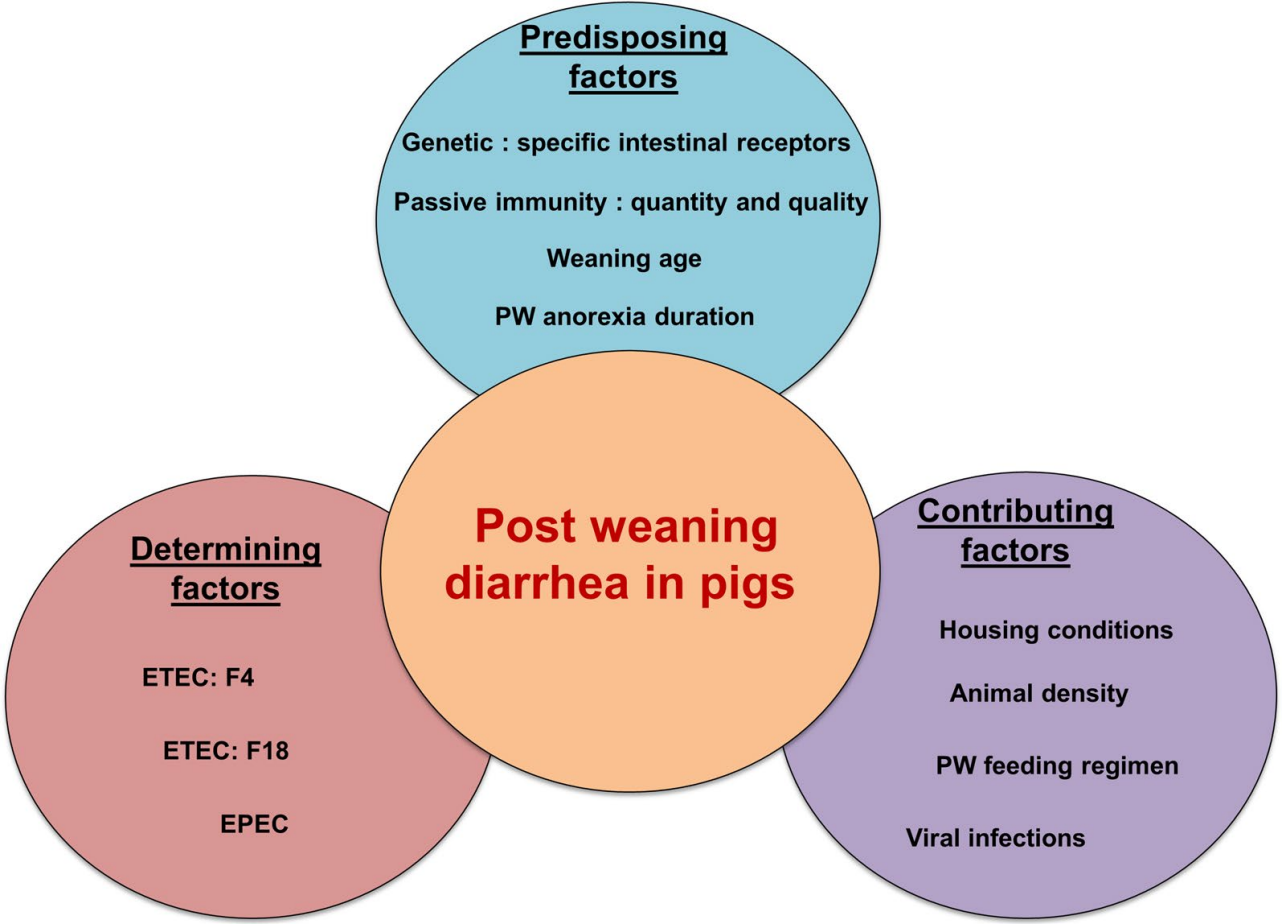
The multifactorial genesis of post weaning diarrhea (PWD) in pigs involves interaction between predisposing, contributing and determining factors. *PW* Post weaning, *ETEC* enterotoxigenic *Escherichia coli*, *EPEC* enteropathogenic *Escherichia coli*

PWD is a multifactorial disease where the exact cause has not yet been identified [26] (Fig. 1). The occurrence of PWD in pigs involves interactions between the sow, piglet, environment, ETEC bacteria and livestock management [27].

#### Predisposing factors

Post-weaning diarrhea is usually associated with the proliferation of one or more strains of β-hemolytic ETEC in the small intestine of pigs, in particular those that express fimbrial adhesins F4 (K88) or F18 [2]. Thus, small intestinal epithelial cell adhesion and subsequent colonization by ETEC is mediated by F4- or F18-specific receptors (F4R or F18R), the existence and function of which are crucial in determining the susceptibility of pigs to ETEC infection [2, 7]. The genetic predisposition of the pig is primordial for the development of PWD [28].

In addition, conditions related to pregnancy and parturition of the sow such as litter size, parity, and postpartum dysgalactia syndrome are significant in the predisposition of piglets to microbial infection [27, 29]. The sow placenta is not permeable to maternal immunoglobulin transport and therefore newborn piglets acquire maternal immunoglobulin from colostrum during the first 24–48 h of life [5]. It was reported that weaning age and pre-weaning health play a key role in the onset of PWD [30]. Moreover, the post-weaning period is a critical phase in the pig’s life when the intestinal immune system is immature, and the sow milk removal, and consequent discontinuation of nutritive intake of the IgA present in this milk, contributes to increase susceptibility of pigs to microbial infections [31]. Indeed, unlike other food animals, the sow’s milk is particularly rich in IgA compared to colostrum [32]. Studies investigating the profitability of weaning pigs at an early age, before 21 days, have further encouraged moving away from this practice to weaning pigs no earlier than 26 days of age to reduce the occurrence of PWD [30, 33]. In the European Union (EU), many pig producers wean piglets at 21 days of age. However welfare legislation encourages weaning no earlier than 28 days of age in the absence of cleaned housing sections to ensure that healthy pigs are transferred into nursery accommodation [34]. Moreover, studies suggest that increasing weaning age reduces stress associated with this period and allows pigs to have a more mature gastrointestinal tract and become increasingly familiar with solid feed during lactation with an improvement in growth performance and in immune response [34, 35].

**Fig. 2:**
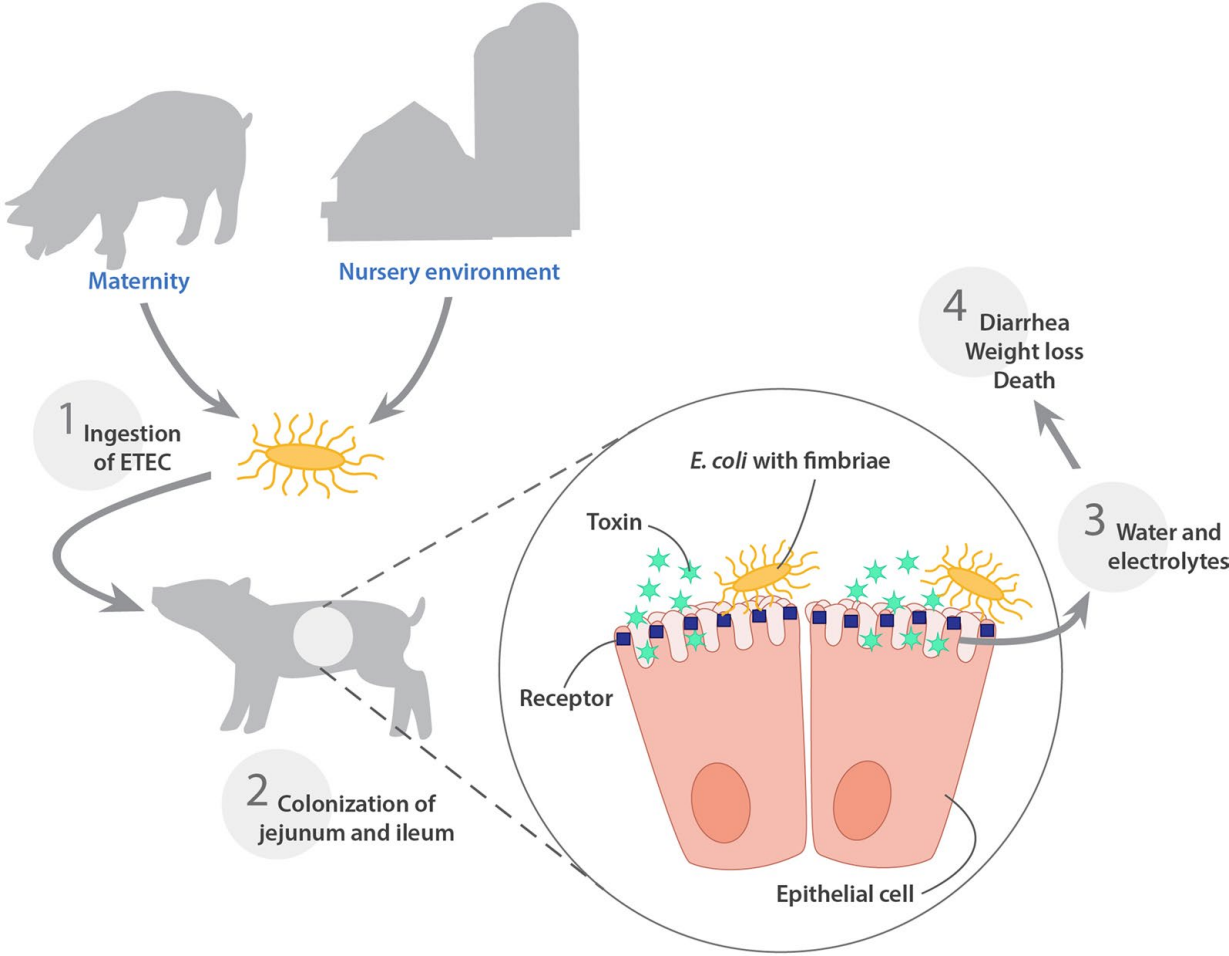
Schematic representation of the steps involved in the pathogenesis of post weaning diarrhea in pigs

Feed intake is usually reduced initially after weaning and the pig may develop anorexia of variable duration and extent between farms, depending on livestock management and the nature of the feed [36]. Madec et al. [30] reported that the low feed intake over the first week after weaning is strongly correlated with the risk of disease occurrence over the post-weaning period. Underfeeding during weaning reduces growth performance of pigs, and contributes to intestinal inflammation and adversely affects villous height and crypt depth [3]. This morphological disruption of the intestinal mucosa promotes the creation of an ideal environment for the multiplication of bacteria such as *E. coli* and allows toxins and bacteria to cross the epithelium as a result of this inflammation [37] (Fig. 2).

#### Contributing factors

Housing factors, population density, parity segregated production and the feeding regimen after weaning play a role in the development of PWD [38].

It is beyond the scope of this review to discuss in detail all the ideal conditions for pig housing during the post-weaning period, but to highlight the most important, as reviewed by Le Dividich and Herpin [39], it is essential to provide the correct environmental temperature, 26–28 °C, to maintain pigs in their thermo-neutral zone. Chilling reduces intestinal peristaltic activity and consequently increases bacterial colonization, and low temperatures in weaner facilities appears to be responsible for a more severe course of PWD [40]. Also, it has been shown that automatic temperature control in the weaners housing reduces considerably the prevalence of PWD [38]. Wathes and Whittemore [41] reviewed several recommendations to prevent pig diseases by appropriate housing and environmental management. These approaches involve avoiding drafts while removing moisture and gases using adequate ventilation. Most often, flat decks are used instead of soiled bedding for weaned piglets; however it was reported that this practice is accompanied by more tail and belly lesions among pigs [42]. Moreover, the removal of manure and soiled bedding on a regular basis is also important to reduce the microbial load on farms.

A contradiction was found in the scientific literature concerning the impact of herd size on the prevalence of PWD in pigs. Indeed, Laine and collaborators reported that in Finland, the increase in pig’s herd size was associated with a higher risk of PWD in pigs [38]. While, in Canada, Amezuca et al. [43] reported that PWD occurred on a variety of farm types and sizes. However, a link between stocking density and PWD was described in pig’s farms in some countries [30, 44]. Mixing piglets from different farms is a common practice in pig husbandry, particularly at weaning. This mixing can result in fighting as the pigs strive to establish dominance relationships, with most aggressive interactions being typically shown during the first few hours after grouping [45]. It has been reported that the hierarchical behaviour among pigs leads to very significant differences in food and water consumption on farms [46]. Production based on segregated sow parities was proposed as a solution to reduce the impact of the social hierarchy. This system of grouping according to the sow’s farrowing rank reduces disease challenge by reducing variation in the immune status of the piglets [47].

It was shown that the prevalence of PWD was higher on farms that fed weaned piglets only twice a day with a restricted amount of feed than on farms that provided more than two meals per day with or without feed restriction [38]. In addition, Amezuca et al. [43] reported that the occurrence of PWD was greater with pelleted feed and inadequate feeder space per piglet in the pen.

A previously mentioned, PWD is a complex disease that may result from interaction between several infectious agents. However, most epidemiological studies have focussed on monitoring the effect of only one pathogen in the occurrence of this disease, and there is inadequate information concerning other relevant enteric pathogens such as viruses and parasites. Some investigations of mixed infections in PWD showed that rotavirus was considered to be an important enteric pathogen in weaned piglets with a prevalence of 77.5%, followed by *E. coli*, coccidia, sapovirus and *Cryptosporidium parvum* with prevalence of 55, 10, 2.5 and 2.5% respectively [48]. In addition, infection by the porcine reproductive and respiratory syndrome virus (PRRSv) results in an impairment of the immune response of piglets, permitting ETEC to cause a septicemia leading to death [49]. However, these data were reported more than 10 years ago and are unlikely to reflect the current epidemiologic situation.

#### Determining factors

ETEC is the most common cause of PWD in pigs. This pathotype is characterized by the production of enterotoxins and adhesins, both essential for disease development. Enterotoxins produced by ETEC may be heat stable [STa, STb, or enteroaggregative *E. coli* heat stable enterotoxin 1 (EAST1)] or heat labile (LT) [2]. Enterotoxin genes are on plasmids of ETEC bacteria and act on the intestinal epithelium of pigs [7].

In pigs, the most frequently found fimbrial adhesins of ETEC are K88 (F4), K99 (F5), 987P (F6), F41, and F18 [40]. F4-positive and F18 ETEC (ETEC: F4 and ETEC: F18) strains represent the major cause of PWD in pigs. F4 are flexible fimbriae that occur as the F4ab, F4ac, or F4ad variant, the F4ac variant being by far the most important type encountered in PWD [50]. The F4 fimbriae mediate bacterial attachment to F4 receptors (F4R), present on the small intestinal brush borders of villous enterocytes allowing ETEC to survive and persist in the small intestine and cause diarrhea [51]. Thus, attachment of ETEC to the pig intestinal mucosa is a crucial step in the pathogenesis and the initiation of PWD. Two antigenic variants of F18 fimbriae exist: F18ab (F107) and F18ac (2134P and 8813). F18ac is commonly associated with ETEC causing PWD, whereas F18ab is often involved in oedema disease [52]. No cross protection between F18ab and F18ac was observed on vaccination against F18 variants [53]. A non-fimbrial adhesin identified as AIDA (adhesin involved in diffuse adherence) has been observed to be associated with ETEC strains recovered from pigs with PWD [54]. In this study, 50.0% of isolates were ETEC-*aidA* ^**+**^. Moreover, it has been demonstrated that the expression of AIDA by a diarrheagenic *E. coli* strain (AIDA-I^+^, STb^+^) was essential for pig’s intestinal colonization and for in vitro bacterial autoaggregation and biofilm formation [55].

Porcine pathogenic *E. coli* involved in PWD typically belong to serogroups O8, O138, O139, O141, O147, O157 and O149, the latter being the predominant serogroup in most countries [56, 57]. The most implicated virotype in PWD is ETEC: LT: STb: F4 [6]. However, O serogroup and virulence gene patterns vary from region to region and over time [2].

Pathogenesis of porcine enteric colibacillosis has been reviewed extensively elsewhere [7, 40, 57]. Indeed, piglets ingest ETEC found in their environment, especially derived from mammary glands of their mother and from the farrowing room or from the pen environment on arrival in the nursery (Fig. 2). These ETEC originate from the gut of piglets with ETEC diarrhea, or subclinical carrier animals at the farm [57]. ETEC bacteria adhere to pig’s small intestinal epithelium, causes an increase of water and electrolytes secretion into the intestinal lumen generated by the release of enterotoxins, and alter the functions of enterocytes by increasing secretion and reducing absorption [7]. Excessive secretion of electrolytes and water leads to dehydration, metabolic acidosis, osmotic diarrhea and possible death [28, 58–60] (Fig. 2). It has been widely reported in the scientific literature that ETEC challenge in pigs was not associated with significant macroscopic lesions or morphological changes in the intestinal mucosa resulting from the toxic activity of ETEC enterotoxins [57, 61, 62]. However, other studies have reported that the necropsy of challenged pigs with ETEC or naturally infected animals, has revealed several lesions such as; dehydration, dilation of the stomach and the small intestines, gastric infarcts in the mucosa of the stomach, and congestion of the mucosa of both the small intestine and the colon [57, 63]. Furthermore, ETEC infections in pigs may also result in a shock syndrome with hemorrhagic gastroenteritis, congestion, renal hemorrhage, and thrombi in the mucosa of the stomach and small intestine [64–66]. Moreover, intestinal ETEC infections in pigs might be associated with secondary septicemia [66] and inactivation of the hemolysin structural gene (*hlyA*) of an ETEC: F4 challenge strain did not decrease the incidence of this septicemia in orally challenged gnotobiotic piglets [66]. Thus, macroscopic lesions of shock syndrome and septicemia related to ETEC infection in the post weaning period in pigs are probably the consequence of the rapid release of bacterial LPS from the pig’s intestine into the systemic circulation [57]. On the other hand, microscopically, ETEC challenge in pigs has been associated with a greater villous atrophy and a large crypt depth reduction in all segments of the intestine of challenged animals [67].

ETEC isolates from pig farms with PWD may show a high frequency of resistance to multiple antimicrobials [1, 68]. Nevertheless, there is no indication that drug resistance enhances the virulence of ETEC, although virulence genes are sometimes associated with drug resistance genes [56].

Porcine attaching and effacing *E. coli* (AEEC) induce intestinal lesions similar to those produced by enteropathogenic *E. coli* (EPEC) in humans, and this pathotype is found in pigs with PWD [40]. These *E. coli* carry the *eae* gene encoding a 94 kDa outer membrane protein (intimin) which is responsible for intimate attachment to epithelial cells. However, the pathogenic significance of porcine EAE positive isolates in weaned pigs is still unknown [2]. Furthermore, identification of porcine EPEC is difficult and many veterinary diagnostic laboratories do not routinely screen for this pathotype of *E. coli*, isolates of which do not usually possess any of virulence factors of classic PWD or oedema strains [40].

### Extent of colistin use in weaned pigs worldwide

The global demand for colistin in agriculture is expected to reach 16,500 tons by the year 2021, this being one of the least expensive classes of antimicrobials available in veterinary medicine in some countries [17]. Thus, the pricing structure makes colistin particularly attractive for use in pig production. Since the inception of its clinical use in 1960, colistin has been used in pig production in many countries for the treatment and prevention of digestive disorders caused by *Enterobacteriaceae*, and even sometimes for growth promotion over long periods, to improve growth rate and feed conversion efficiency in pigs [18, 69, 70]. In certain countries such as Canada, where colistin has not been approved for use in pigs, a rapid increase in resistance of ETEC to a wide range of antimicrobials has prompted the use of colistin in weaned pigs under the veterinarian’s responsibility [71]. However, current data on the total quantities of colistin used in pigs worldwide have been difficult to obtain accurately [70]. Some data, for example in Denmark, indicate that the use of colistin for the treatment of sows increased between 2002 and 2008 [72]. Of the two forms of colistin commercially available, colistin sulfate (CS) and colistin methanesulphonate sodium (CMS), and only CS is approved in pig production in some countries [18]. Usually it is administered orally in the drinking water at the dose of 50,000 IU/kg body weight every 12 h for 3 or 5 days [25]. Colistin is mostly used in monotherapy in pigs, although it may be combined with other antimicrobials, such as amoxicillin, for the treatment of PWD [25, 73].

#### Colistin use in post weaning diarrhea on farms

Due to its activity directed against GNB, colistin is widely used for the control of PWD in pigs [10, 74]. Two surveys conducted in pig farms in Belgium, in 2006 [73], and 2012 [74] confirmed that colistin was the most frequently used antimicrobial for the control of PWD, being mostly used prophylactically. However, colistin was underdosed in 90 and 53% of the cases, in the first and the second survey respectively. In Germany, it was reported that intestinal diseases in weaners were commonly treated with colistin, pigs being treated 9.7 days (median) per 100 days with this antibiotic, although tetracycline and tylosin were also used in approximately equal amounts [75]. In a study in France, it was reported that 90% of pig farms used colistin during the post-weaning period [10]. In Vietnam, a survey conducted on pig farms representing three different animal production systems (farm household, semi-industrial and industrial) showed that colistin was the most commonly used antimicrobial for prevention and therapy of gastrointestinal disorders in pigs [76].

It has been reported that China is the country with the greatest use of colistin in pigs worldwide [17], although we did not find any reports in the literature on surveys of colistin use in this country in the post-weaning period. Overall, colistin is widely used in the management of the PWD, with a lot of differences between countries in terms of quantities used and modality of administration [18].

#### Microbiological and clinical outcomes of colistin use in controlled conditions

Most of the recent studies conducted in pigs have used CS in experimental conditions for the control of diarrhea in the post-weaning period (Table 1). Several of these studies were performed to examine the effectiveness of alternative substances to colistin in the treatment of PWD [77, 78].

**Table 1:** Microbiological and clinical outcomes of monotherapy with colistin in pigs

Bacterial agents/condition	Dose per day	Duration (days)	Sample type	Reduction *in E. coli* (log cfu/g)^c^	Performance (ADG, g/day)	References
*E. coli* K99/experimental PWD	300 mg/kg of diet	7	Ileum	6.55	122^b^	[154]
			Cecum	6.63		
*E. coli* K99/experimental PWD	300 mg/kg of diet	10	Ileum	2.3	128^a^	[77]
			Cecum	3.2		
Weaned pigs (clinically healthy)	200 mg/kg of diet	7	N/A	N/A	229^a^	[155]
ETEC mixture/experimental PWD	200 mg/kg of diet	21	Ileum	1.54	292^b^	[156]
			Cecum	1.65		
			Colon	0.65		
ETEC mixture/experimental PWD	2.5 mg/animal (Oral-Water)	21	Fecal samples	3	283^b^	[78]
Weaned pigs (clinically healthy)	40 mg/kg of diet	14	Ileum	N/A	142.2^a^	[81]
			Cecum			
			Colon			
*E. coli* K88/experimental PWD	4.8 mg/kg (Oral-Water)	5	Fecal samples	4	214^a^	[28]
*E. coli* K88/experimental PWD	9.6 mg/Kg (Oral-Water)	5	Fecal samples	4	N/A	[28]
Weaned pigs (clinically healthy)	172.8 mg/kg of diet	14	Fecal samples	4.5^d^	N/A	[80]

*PWD* post-weaning diarrhea, *ADG* average daily weight gain, *N/A* not available, *ETEC* enterotoxigenic *Escherichia coli*

^a^Not statistically significant compared to the control group

^b^Statistically significant compared to the control group

^c^Reduction compared to the control group

^d^log cfu of *Enterobacteriaceae*/g

It is often difficult to compare results between studies, because of the variability in the dose of CS used, treatment duration, and the experimental design of the study. In Table 1, we have summarized the main results reported in the literature concerning fecal *E. coli* shedding and pig performance following oral CS treatment. Several studies have also followed histological (i.e. intestinal mucosa morphology) and biochemical (e.g. d-lactate, nitric oxide, xylose, etc.) parameters subsequent to CS use in the post-weaning period in pigs [78, 79]. In order to evaluate the effect of colistin on fecal *E. coli* shedding, bacterial quantification was performed in most studies using culture methods [28, 77], whereas other studies used real-time PCR [79, 80]. Furthermore, the oral use of a high dose of colistin in healthy piglets was not associated with a major perturbation in the pig gut microbiota as demonstrated by a high-throughput sequencing method [80].

Although colistin has been used in some studies to promote animal growth, data were not conclusive to support the effectiveness of this practice [81]. In this study, no difference was observed between the CS treated and the control group in terms of average daily gain per day (ADG/day) [81]. Also, the economic benefits of antimicrobial growth promotion in modern farms have been questioned [82], the benefit of this use being associated with poor hygiene on farms.

### Alternative strategies to colistin for post-weaning diarrhea control

Reduced colistin usage in livestock and particularly in swine is highly promoted worldwide and is required in Europe as a public health measure to reduce colistin resistance spread, and to prevent the loss of polymyxins effectiveness in human medicine [25]. Furthermore, concurrent treatment with colistin in piglets was associated with the isolation of resistant bacteria from the earliest days of treatment [28]. Almost all studies conducted on isolates from pigs worldwide to screen *mcr*-*1* gene presence in enterobacterial species reported that colistin resistant isolates harboring this gene also showed resistance to one or several classes of antimicrobials conventionally used in swine such as: aminoglycoside, sulphonamide, trimethoprim, tetracycline, quinolone, lincosamide, β-lactam, and third generation cephalosporin [83–86].

However, to ensure swine welfare, productivity and reduced mortality associated with PWD, alternatives to colistin and other antimicrobials, especially those of critical importance for human health, are essential in pigs. There is a major debate over the terminology ‘alternative to antibiotics’ because we do not propose substances with antibacterial activity but rather substances that act on bacteria indirectly, either by stimulating the host immune system, by the release of substances that have antibacterial activity or by improving the host gut health and consequently growth performance [87]. Thus, we will use the terminology «strategies» or «measures» to describe alternatives to antimicrobials. Due to the multifactorial etiology of PWD, finding case-specific preventive measures against this disease is a challenge for both researchers and veterinarians. Here we give an overview of these preventive strategies, focusing on the most practical and promising ones for the control of PWD in pigs.

#### Preventive measures

In the literature, many alternatives to antimicrobial usage in food-producing animals have been reported and discussed [87–90]. The most promising way to mitigate the development of colistin resistance is to reduce the use of antimicrobials at the farm level (Table 2). There are documented relationships between housing conditions and incidence of PWD in pig herds; Madec et al. [30] claimed that prevention of PWD disorders could be based solely on the control of zootechnical conditions. Moreover, stocking density reduction could be considered as a paramount strategy to decrease occurrence of PWD as well as other diseases in pigs [91]. Thus, improvement of breeding conditions in pig farms is a crucial measure to reduce the susceptibility of animals to microbial infections and consequently to reduce the use of antimicrobials in pig production [28]. The management strategies around weaning should focus on measures that avoid any kind of stress for pigs. These measures include preventing the spread of infection, providing the pigs with good thermal comfort, giving them adapted feed and allowing access to this feed for all pigs.

**Table 2:** Preventive strategies to reduce the use of antimicrobials during the post-weaning period

Strategies	Benefits	Limitations	References
Control of housing environment and improved biosecurity	Very effective approach	Significant cost	[28, 30]
	Significantly reduces PWD occurrence	Extreme weather conditions in some countries	
	Reduces the use of antimicrobials in farm	Acceptability of farmers to change some management techniques	
	Sustainable approach	Financial support is required	
Diet management (reducing the amount of soybean)	Reduces the severity and frequency of PWD and oedema disease	Growth retardation	[31]
		Increase production	
	Reduction of histological changes in intestinal crypt and villi	Considerable controversy between studies	
Communicative advisory tools for pig farmers	Improving breeding management	Requires a lot of field work	[94]
	Farmers feel concerned by the problem of antibiotic resistance	Farmers worried mostly about infectious diseases and financial issues	
	Raised awareness and responsibility	Financial bonus is required	
Laboratory diagnosis to confirm etiology of PWD	Avoid the use of antimicrobials to treat viral diarrhea	Significant cost	[95]
	Allows an appropriate choice for antibiotics	Lack of rapid diagnostic techniques	
Policy measures	Reduce the sale and the use of antimicrobials on farm	Requires penalties	[94]
	Reduce self-medication	Financial bonus is required	
Immunoprophylaxis: Live attenuated and live wild type avirulent *E. coli*	Specific protection against ETEC: F4 or F18	Interference with the lactogenic immunity of piglets	[97]
	Easy to administer on farms (drinking water)	Absence of cross-protection between F18ab strains	
	Reduces antimicrobial use in the PW period	Limited availability in some countries	
	Marketed in swine		
Immunoprophylaxis: Subunit vaccines (purified F4 fimbriae)	A powerful oral immunogen	The proposed immunization procedure required large quantities of F4	[8]
	Leads to a specific mucosal immune response	Antigen degraded by the pH of the stomach and by digestive enzymes	
	Leads to a significant reduction in fecal excretion of ETEC: F4	Usually required mucosal adjuvant such as Cholera toxin	
Breeding of resistant pigs	Very effective approach	Expensive process	[2]
	Greatly reduces the total amount of antimicrobials used on farms	Lack of techniques for a large-scale selection	
	Reduces the selection pressure	Development of other adherence mechanisms	

*PWD* post weaning diarrhea

Considerable research has been performed into developing diets for weaners and there is now a range of high quality diets that are readily digested by the early-weaned pig [31]. The main purposes of these diets are to achieve high post-weaning feed intakes and minimize duration of post-weaning anorexia and consequently growth retardation. It has been reported that the presence of some ingredients in the feed for weaners, such as soybeans, seems to favor the occurrence of PWD [92]. This could be due to the presence of trypsin inhibitors or antigens inducing a localized immune response [2]. Furthermore, it was shown that soya bean meal (SBM) reduced duodenal specific activities of most intestinal enzymes and increased crypt depth in pigs [93]. Thus, such ingredients should be avoided in feed of early-weaned pigs. In addition, feeds with decreased protein content and the addition of organic acid to reduce gastric pH were found to decrease *E. coli* colonization and to minimize PWD prevalence [31].

The scientific community increasingly recognizes the importance of communication and awareness among farmers in relation to antimicrobial resistance, as reflected by the growing number of publications in this area in recent years [18, 94]. This suggests that farmers’ perceptions, and the factors affecting their behaviour, need to be better understood if effective measures associated responsible and prudent use of antimicrobials are to be implemented successfully.

Moreover, effective diagnostic tools are essential for veterinarians to confirm the bacterial etiology of PWD and to determine the antimicrobial susceptibility of the identified bacterial strain. The laboratory diagnosis is particularly important in PWD to avoid the inappropriate use of antimicrobials. DNA-based molecular detection methods such multiplex PCR based on the detection of ETEC virulence genes are rapidly becoming part of the routine laboratory diagnosis of PWD, and these genes are used as a biomarkers of ETEC strain [7, 58].

In several countries, implementation of financial penalties for high antimicrobial users is proposed as a method to reduce antimicrobial usage and pig farmers would receive a financial bonus when they use alternative methods or when they greatly reduce antimicrobial use on their farms [94]. Vaccination seems to be an effective approach to reduce the occurrence of PWD and to reduce infection pressure and increase immunity in the pig population [2]. Several studies conducted in pigs confirm a reduction of antimicrobial usage after vaccination [95]. In fact, vaccination against the porcine proliferative enteropathy caused by *Lawsonia intracellularis* reduced the need for therapeutic oxytetracycline administration in Danish pigs [96]. Live attenuated and wild type avirulent *E. coli* vaccines appear to be promising for the control of ETEC infections and live vaccine against ETEC: F4, is now available in Canada and Europe [97]. This vaccine is added to the drinking water and recommended for the vaccination of healthy weaned pigs of 17 days or more. Clinical studies confirmed that administration of this vaccine significantly reduced intestinal colonization by virulent ETEC: F4 and the accumulation of fluid in the intestines after an experimental challenge [98]. The immunity in piglets begins 7 days after oral vaccination, however, since PWD caused by ETEC: F4 occurs shortly, in the first week, after weaning, an immune trough may exist in the first days after weaning during which the pigs are not protected [97]. Thus, the time of the administration of this vaccine should be adjusted. In addition, clinical trials of vaccination against ETEC: F18 has been carried out in pigs. Genetically susceptible pigs were vaccinated orally on three consecutive days, beginning 10 days before weaning with a live F18ac-positive *E. coli* vaccine [53]. In this study, a significant rise in F18ac-specific serum IgA and a 3 Log CFU decrease in fecal shedding of the F18ac-positive challenge strain was observed compared to the unvaccinated group. However, this vaccine did not induce protective immunity against ETEC: F18. On the other hand, it was shown that a minor subunit of F18 (FedF) alone or genetically fused to F4 FaeG subunit or conjugated to F4 fimbriae induced protective anti-F18 antibodies in pigs [99]. In general, the success of a vaccine against PWD depends largely on the identification of the most prevalent ETEC pathotype present in the farm, resulting in matching of the appropriate protective antigens with the adhesin produced by the ETEC present on the farm, and administering it at the optimal time [7]. For vaccines consisting of live F4 or F18ac-positive *E. coli,* it is often recommended to vaccinate suckling pigs to obtain a strong mucosal immunity production, IgA, before weaning. However, our knowledge is very limited about the effect of maternal antibodies on the survival of these vaccine strains in the intestine of pigs of this age. Also, there is no cross protection against ETEC strains expressing a different fimbria or toxin. Recently, plant-based vaccines for protection of pigs against ETEC were investigated. A rice-based cholera vaccine expressing the choleratoxin (CT) subunit B (CTB) (MucoRice-CTB) was tested in pigs for protection against LT-ETEC infection [100]. CTB-based vaccines can target not only F4-type but also F18-type ETECs, and this vaccine also induced maternal CTB-specific IgG and IgA in the colostrum and milk of sows after farrowing. CTB-specific antibodies were also secreted into the gut lumen of weaned pigs and reduced intestinal loop fluid accumulation upon ETEC challenge, indicating a protective effect of this vaccine against ETEC diarrhea [100]. However, the cost of these vaccines is very high and, unlike open-air farming, the production of transgenic plants for biotherapeutic use is very demanding. Moreover, the procedures for manufacturing and processing of plant-based pharmaceuticals are not well defined. Thus, a large-scale production of these vaccines not envisaged, at least in the near future. Current progress in the development of subunit vaccines against ETEC associated with diarrhea in humans and animals has been reviewed extensively elsewhere [97, 101]. However, none of these subunit vaccines has been marketed in swine.

The selection of animals genetically resistant to ETEC F4 and/or F18 is considered as a radical solution to eliminate the PWD in a swine herd. However, progress in this area is very limited or even non-existent. Pigs that are resistant to ETEC: F4 and/or F18 do not express intestinal receptors for these fimbrial types [2]. The expression of these receptors is genetically determined and inherited in a dominant way and the loci controlling F4R and F18R expression are located on separate chromosomes. The gene underlying resistance to F4ab/ac ETEC has been assigned to porcine chromosome 13, whereas the F4ad ETEC receptor is located on another chromosome that was not identified [102]. A PCR–RFLP test has been developed to allow genotyping for F4ab/ac ETEC resistance/susceptibility [103]. Three different genotypes were observed and were identified as resistant (RR), susceptible heterozygote (SR) and susceptible homozygote (SS). However, it cannot be predicted if additional types of adhesive fimbriae or new variants of known types will emerge which could bind to yet unidentified receptors and could cause outbreaks of diarrhea and mortality in the nursery [2]. It is difficult to understand the reasons behind the non-exploration of the genetic breeding for ETEC resistant pigs to reduce economic losses associated with PWD and to reduce the use of antimicrobials on farms. It was shown in an early study that F4 susceptible piglets tend to have better growth performance then F4 resistant ones [104]. Also, heterozygous F4R^−^ piglets are not passively protected from infection by ETEC: F4 strains [105].

#### Feed additives

In pigs, PWD can be controlled using various preventive strategies without using antimicrobials (Table 3). Feed supplements such as zinc oxide, organic acids, pre-probiotics, synbiotics, dehydrated porcine plasma, antimicrobial peptides, specific egg yolk and bacteriophages [31, 89, 106–110] have been used in weanling pigs to enhance growth, feed efficiency and to reduce PWD. Here we give an overview of these feed strategies, focusing on the most used practices showing clinical effectiveness in reducing symptoms of PWD and ETEC attachment to enterocytes.

**Table 3:** Benefits and limitations of the major alternative feed strategies for the control of post weaning diarrhea (PWD) in pigs

Strategies	Benefits	Limitations	References
Zinc oxide	Inhibition of bacterial adhesion to the intestinal mucosa	High levels increased PWD	[112, 115]
	Stimulated growth rate	Soil heavy metal contamination	
	Maintained intestinal mucosal integrity	Bacterial resistance	
	Modulated immune functions	Co-resistance	
Organic acids	Decreased pH in the stomach	Exact modes of action still unknown	[108]
	Improved growth performance	Anti microbial activities is different between acids	
	Reduced PWD		
Prebiotics, probiotics and synbiotics	Improved intestinal health	Sometimes contradictory studies on their effectiveness	[127, 130]
	Improved growth performance	Lack of information on the potential synergism between pre- and probiotics	
	Reduced ETEC: F4 attachment to the ileal mucosa		
	Reduced diarrhea		
Spray dried plasma (SDP)	Improved growth performance	High cost	[111]
	Reduced incidence and severity of diarrhea	Required rigorous control during the preparation process	
	Reduced the markers of intestinal inflammation	Potential source of pathogens?	
	Maintained mucosal integrity		
Antimicrobial peptides (AMPs)	Improved growth performance	The pharmacokinetics in vivo is unknown	[89, 139]
	Decreased diarrhea	Bacterial resistance	
	Reduced the markers of intestinal inflammation		
	Enhance immune function		
	Cocktails of AMPs might be used to mitigate selection for resistance		
Specific egg yolk antibodies	Improved growth performance	High cost	[111]
	Decreased diarrhea	Antibodies are sometime not specific against the infecting ETEC strains on farms	
	Maintained intestinal mucosal integrity		
Bacteriophages	Reduced *E. coli* mucosal adhesion	Narrow spectrum of activity	[144]
	Maintained intestinal mucosal integrity	Development of bacterial resistance	
	Decreased diarrhea	A combination of phages is needed	

Zinc oxide: it has been shown that the addition of zinc (Zn) as zinc oxide (ZnO) at the levels of 2400–3000 ppm in pig feed was effective in the reducing of PWD and mortality and in improving growth performance in weaned pigs [111, 112]. However, Amezcua and collaborators [1] reported an important proportion of farms with PWD occurrence using high levels of ZnO. Also, several studies reported an increased proportion of *E. coli* isolates resistant to tetracycline and sulfonamides in pigs fed with high zinc doses [113, 114]. This may explain why antimicrobial resistance persists even in the absence of antimicrobial exposure [115, 116]. Moreover, the use of high zinc levels in pig feeds has led to heavy metal contamination in the soil, raising environmental concerns [115]. Recently, Bouwhuis et al. [117] reported that organic zinc [zinc methionine (ZnM)] could be used as a substitute for the inorganic zinc (ZnO) in the pig diet. In fact, organic zinc can be supplemented in lower doses (up to 500 mg/kg feed) compared to ZnO [117]. In this study, the inclusion of ZnM resulted in improved faecal scores and the intestinal architecture compared to that observed in pigs supplemented with ZnO.

Organic acids such as citric, fumaric, lactic, propionic, benzoic and formic acids showed beneficial effects in the pig gastrointestinal tract. In fact, the use of organic acids in weaned piglets was associated with a reduction of stomach pH [118]. With this effect, organic acids generate a hostile gastric environment for bacterial survival. Moreover, organic acids promote the conversion of pepsinogen into pepsin in the stomach of pigs, and promote the activity of this enzyme [108]. On the other hand, decreasing the intestinal pH is probably not a primary effect of feeding organic acids in pigs. Indeed, Risley et al. [119] reported a non significant decrease in the pH of the small intestine in 3-week-old weanling pigs fed with a diet supplemented with 1.5% fumaric or citric acid. Addition of organic acids to weaned pig diets improved growth performance and health [31] as well as the local immunity in the jejunum epithelium [120]. It was reported that regardless of the organic acids used in the feed, these compounds reduced the incidence and severity of diarrhea in pigs, and improved the performance of the treated group compared to that of the negative control group [121].

Prebiotics are selectively fermented components of feed, indigestible by the host animal, that modulate the gut microbiota to benefit host health. Resulting effects include the stimulation of short-chain fatty acid (SCFA) production and the proliferation of *bifidobacteria* and lactic acid bacteria such as *Lactobacillus* spp. and *Bifidobacterium* spp. [122, 123]. Common prebiotics include inulin and oligosaccharides such as galactooligosaccharides (GOS) and fructooligosaccharides (FOS) [124]. Pigs fed with chito-oligosaccharides (COS) showed better overall intestinal health (based on villi height), improved performance (measured by body weight gain and feed conversion ratio) and higher *Lactobacillus* counts than those found in control pigs or pigs receiving diets supplemented with chlortetracycline [125]. Also, fermented ingredients, such as non-starch polysaccharide hydrolysis products of soybean meal (SBM) in weaned pig feed, were found to interfere with attachment of ETEC to enterocytes and were beneficial in maintaining fluid balance during ETEC infection [126]. It was shown that the prebiotic β-galactomannan (βGM) inhibited the in vitro adhesion of ETEC on the cell surface of porcine intestinal IPI-2I cells, and decreased the mRNA ETEC-induced gene expression of pro-inflammatory cytokines such as TNF-α, IL-6, GM-CSF and chemokines on intestinal IPI-2I cells [127].

Probiotics such as lactic acid bacteria, *Bacillus* and yeasts are live microbial feed supplements [122]. Probiotic bacteria have also been shown to produce antimicrobial molecules, such as bacteriocins, and to inhibit the production of bacterial toxins or the adhesion of pathogens to the intestinal mucosa [123]. Several studies demonstrated that pre-treatment with certain probiotics, such as *L. rhamnosus*, was effective in reducing diarrhea in experimental ETEC: F4 PWD in pigs, possibly via the modulation of the intestinal microbiota, enhancement of intestinal antibody defense, and regulation of production of systemic inflammatory cytokine [128]. Recently, Lane et al. [129] reported that *L. acidophilus* supplementation (0.2%) in the weaned pig diet resulted in higher *Lactobacillus* counts and lower *E. coli* counts, as well as an increase in ADG and the average daily feed intake in supplemented pigs compared to the basal diet pigs. A *Bacillus licheniformis* and *Bacillus subtilis* spore mixture (BLS-mix) was effective in preventing loss of intestinal epithelial barrier integrity after a challenge with ETEC: F4 in experimental PWD [130]. In addition, it was shown that the feeding of pigs with live yeast *Saccharomyces cerevisiae* enhanced their growth and reduced the duration and the severity of PWD caused by ETEC [131]. It has been demonstrated that the administration of a mixture of two probiotics, *Pediococcus acidilactici* and *Saccharomyces cerevisiae boulardii*, in the feed of challenged weaned pigs reduced ETEC: F4 attachment to the ileal mucosa in comparison with the group treated with chlortetracycline and tiamulin [103].

Synbiotics refers to a combination of probiotic and prebiotic approaches; it is possible that a prebiotic that confers gastrointestinal health benefits could selectively increase the population and/or activity of probiotics in the gut [132]. Synbiotics can be either complementary or synergistic. Complementary synbiotics consist of a probiotic and a prebiotic selected independently to confer benefits to the host. On the other hand, synergistic synbiotics are comprised of a prebiotic chosen specifically for the selected probiotic to potentiate its effect in the gut [133]. It was shown that the combination of raw potato starch and a probiotic had a beneficial effect on pig growth performance and resulted in a reduction of diarrhea and increased microbial diversity in the gut of weaned pigs challenged with an ETEC: F4 strain [134]. Also, Guerra-Ordaz et al. [135] showed that following a challenge of pigs with pathogenic *E. coli* (O149:K91:H10), administration of a prebiotic oligosaccharide, lactulose, in the feed resulted in improved weight gain, increased lactobacilli and the proportion of butyric acid in the colon, and less inflammation due to a reduction of the pig major acute-phase protein (Pig-MAP) in serum. Administration of *Lactobacillus plantarum* in the feed promoted lactobacilli growth, modulated fermentative activity, reduced inflammation, and improved intestinal mucosa function and showed a tendency to reduce diarrhea. The application of a synbiotic diet resulted in the benefits of both diet regimes, thus being an example of a complementary synbiotic [135].

Spray dried plasma (SDP) is a protein rich product obtained from the industrial fractionation of blood from healthy animals [106]. It was shown that addition of SDP to the feed improved growth performance, and protects pigs against ETEC: F4 infection by reducing the intestinal expression of inflammatory cytokines such as TNF-α and interleukin-8 and maintaining mucosal integrity, and enhancing specific antibody defense [111]. Spray dried plasma (SDPP) of porcine origin has been pinpointed as a potential source for the coronavirus in a recent epidemic of porcine epidemic diarrhea (PED) [136]. Thus, spray-dried chicken plasma (SDCP) has been evaluated as a replacement for SDPP in weaned pigs. Indeed, the effect of SDCP on serum biochemistry, intestinal barrier function, immune parameters, and the expression of intestinal development-related genes in piglets was similar to SDPP [137]. Nevertheless, a study has provided evidence that PED virus is inactivated during the SDPP production process [138].

Antimicrobial peptides (AMPs) are small molecules constituting an important part of the innate immune system. They may present antibacterial, antifungal, antiparasitic, and antiviral activities, and are increasingly of interest as alternatives to classic antibiotics [88]. AMPs such as lactoferrin, cecropin, defensin, plectasin and bacteriocins showed beneficial effects on growth performance, nutrient digestibility, small intestinal morphology and gut microbiota in pigs [89]. Available data on the effect of AMPs on swine health and especially in the control of PWD have been reviewed extensively elsewhere [89, 139]. Antimicrobial lactoferrin peptides are one of the most commonly used AMPs in pig feeds. More recently, it was shown in a murine model of intestinal inflammation that treatment with porcine lactoferrin-derived peptide LFP-20 was effective in the prevention of histological damage, the inflammatory response and the disruption of tight junction structure induced by LPS in the intestine [140]. Colicins, a class of bacteriocins produced by *E. coli* and closely related species, have been shown to inhibit the activities of ETEC: F4 and F18 strains in vitro and in vivo, and improve the growth performance, reduce the incidence of PWD and the expression of the IL-1β and TNF-β genes in ileal tissues of pigs [141]. On the other hand, resistance to AMPs has been observed in vitro in GNB such as *E. coli* [142]. Thus, the use of AMPs in pig farms needs careful and controlled implementation to limit possible resistance development and cocktails of AMPs might be useful to mitigate selection for resistance [88].

Specific egg yolk antibodies: The chicken egg yolk is a source of large quantities of relatively inexpensive IgY antibodies [2]. Several studies reported that specific chicken antibodies provide protection against ETEC infections in pigs [111]. Despite the effectiveness of this practice, we have not found in the recent literature (last 5 years) any studies evaluating the use of specific egg yolk antibodies in PWD control. This is probably the consequence of the non-profitability in pig production of this practice, or the lack of protection against ETEC challenge or PWD occurrence, possibly because the antibodies contained in the eggs are not specific against the infected ETEC strains present on the farm [143].

Bacteriophages are highly species-specific viruses that can infect and kill bacteria. They have been widely evaluated in clinical trials to treat bacterial infections in pigs as an alternative to antibiotics use [144]. Recently, it was reported that dietary supplementation with bacteriophages for the treatment of PWD caused by an ETEC: F4 strain in an experimental model, was effective in reducing rectal temperature, faecal consistency score, *E. coli* adhesion score in the ileum and caecum, and villous height/crypt depth ratio (VH/CD) in the duodenum and jejunum [145]. However, there are several disadvantages associated with the use of phage therapy in swine. Phages have a narrow spectrum of activity directed against a limited number of bacteria and the possible development of bacterial resistance against phages has to be considered [144]. To overcome the narrow spectrum of activity, some recent studies have reported beneficial effects of a bacteriophage cocktail used in the feed for weanling pigs. This combination resulted in enhanced growth performance and gut health of pigs, although the combination of phages with probiotics did not show any additional effect [109]. Some authors have considered that the development of phage-resistant bacteria could be positive for the host [146]. In fact, resistance to phages can reduce the fitness of the bacteria and could thereby impair their competitive capacity and consequently their ability to colonize the intestinal mucosa of the host [146].

Others: Several studies have documented a significant improvement of weight gain, and feed conversion, as well as the reduction of the incidence, severity and duration of diarrhea in weaned pigs fed diets supplemented with substances such as: exogenous enzymes [147], milk products [148], clay minerals [149], and medicinal plants [150]. Although many peer-reviewed studies discussing these substances are available in the scientific literature, most of the clinical studies were performed in experimental conditions. More research is needed to evaluate the potential effectiveness of these substances under field conditions for the control of PWD in pigs.

#### Results of comparative studies

Several studies have been carried out in experimental conditions to assess the effectiveness of alternatives to colistin for the control of PWD in pigs (Table 4). Here, we give an overview of studies published in 2015 or 2016.

**Table 4:** Effects of colistin compared to alternative measures for control of post weaning diarrhoea (PWD) in pigs

Trials	ADG (g/day)	Ileum villus height (μm)	Ileum crypt depth (μm)	*E. coli* (log 10 CFU/g)	Diarrhea	References
Study 1: HP	d0–35	d35	d35		d0–21^c^	[151]
Hop β-acids^e^ (360 mg/kg)	441^a^	337	214	NA	1.51	
Colistin sulfate (40 mg/kg)	425^a^	366	230	NA	1.51	
Control	387^b^	349	219	NA	1.72	
Study 2: HP	d21	d21	d21	d21		[152]
Two *Macrocephala* flavored powder (3000 mg/kg)	NA	121	66.30	7.93^a^	NA	
Colistin sulfate (300 mg/kg)	NA	107	57.63	6.48^a^	NA	
Control	NA	120.49	64.75	6.63	NA	
Study 3: HP	d1–21	d21	d21	Ileum d21^f^	d1–7^c^	[79]
Recombinant plectasin (Ple) (60 mg/kg)	311.43^a^	227.69	95.53	6.61	10.48	
Colistin sulfate (60 mg/kg)	333.57^a^	195.57	88.48	5.86	8.57	
Control	193.10^b^	160.45	105.82	6.29	36.19	
Study 4: HP	d0–14				d0–14	[81]
Medium-chain triglyceride (MCT) (3000 mg/kg)	141.2	NA	NA	NA	0.91	
Colistin sulfate (40 mg/kg**)**	142.2	NA	NA	NA	0.91	
Control	130.7	NA	NA	NA	1.01	
Study 5: HP	d28–56	d42	d42		d28–56^d^	[157]
Freshwater microalgae *Chlorella vulgaris* (1000 mg/kg)	395	435	278	NA	24^b^	
Colistin sulfate (20 mg/kg)	400	440	283	NA	34^a^	
Control	393	415	299	NA	36^a^	
Study 6: CP		d1 post challenge^e^	d1 post challenge^e^			[158]
Live yeast (5 × 10^10^ CFU/kg)	NA	322	246	NA	NA	
Colistin sulfate (1000 mg/kg)	NA	334	236	NA	NA	
Control	NA	294	199	NA	NA	

Live yeast: *Saccharomyces cerevisiae*

*HP* healthy pigs, *CP* challenged pigs, *NA* not available

^a,b^Values within a row with different superscripts differ significantly at *P* < 0.05

^c^Diarrhea occurrence was calculated as the proportion of days in which pigs showed clinical signs of diarrhea

^d^Number of pig days with diarrhoea score ≥2

^e^Jejunum

^f^log (copies/g)

Several recent experimental studies have now shown that some alternatives (Table 4) resulted in similar or superior clinical outcomes compared to colistin for improving growth performance and intestinal integrity and in reducing of incidence of diarrhea in weaned pigs. In fact, no difference was observed in growth performance of weaned pigs supplemented with hop β-acids (120, 240, or 360 mg/kg) or colistin (40 mg/kg) during a trial period of 35 days [151]. Moreover, the supplementation of weaned pigs with two *Macrocephala* flavored powder (3000 mg/kg) increased significantly villus height in the duodenum and jejunum compared to that observed in colistin (300 mg/kg) supplemented pigs [152]. However, these studies (Table 4), were conducted in experimental conditions and in most cases in healthy weaned pigs. Thus, further research is needed to demonstrate the stability and the efficacy of such alternatives (probiotics, AMPs, medicinal plants) in field conditions as well as the safety of these substances in animals and for consumers. Also, work is needed to optimize the doses of these substances to incorporate in the feed to ensure their effectiveness in PWD control. The financial cost and the ease of administration of such alternatives are the other important criteria that should be taken into consideration in pig production.

#### Limits and perspectives

A long and growing list of compounds have been tested for their ability to replace colistin or other antibiotics for the control of PWD in pigs. However, it is difficult to identify a single “ideal” solution for PWD management. Also, as was discussed above, PWD is a multifactorial disease and the exact overall etiology has not yet been fully elucidated, making it difficult to choose suitable alternatives. Moreover, the most of these alternatives produce inconsistent results regarding their effectiveness in field conditions [107]. Oral administration of specific-antibody-containing egg yolk, or SDP to weaned piglets showed in some cases no protection against ETEC strains or PWD outcomes, likely because the contained antibodies were not specific against the infecting ETEC strains present on the farm [2]. The composition of plant extracts, organic acids and probiotics is complex and knowledge regarding their mechanisms of action is poor, resulting in variable results and safety risks [87]. Synergy mechanisms of probiotics and prebiotics are not very well known nor well studied [133]. Although AMPs and bacteriophages helped in the treatment of PWD, the bacterial resistance risk, the high cost and the narrow antibacterial spectrum of these alternatives reduce their practical use on farms [88]. Vaccination is one of the most promising strategies for the control of PWD in pigs both in terms of preventive ability and cost-effectiveness [97]. The control of production parameters (temperature, ventilation, density, sanitation, biosafety, improvement of feed quality) are crucial factors for the control of PWD and the reduction of the use of antimicrobials during the post-weaning period [28]. However, the improvement of farm conditions and management requires investment and awareness of pig farmers. Furthermore, the use of regular diagnostic testing is crucial to ensure an appropriate choice of the antimicrobial and to monitor its effectiveness on farms. Thus, efforts to improve microbiological laboratory detection methods are of paramount importance to help the veterinarian to act rapidly at an early stage of the disease [153].

**Fig. 3:**
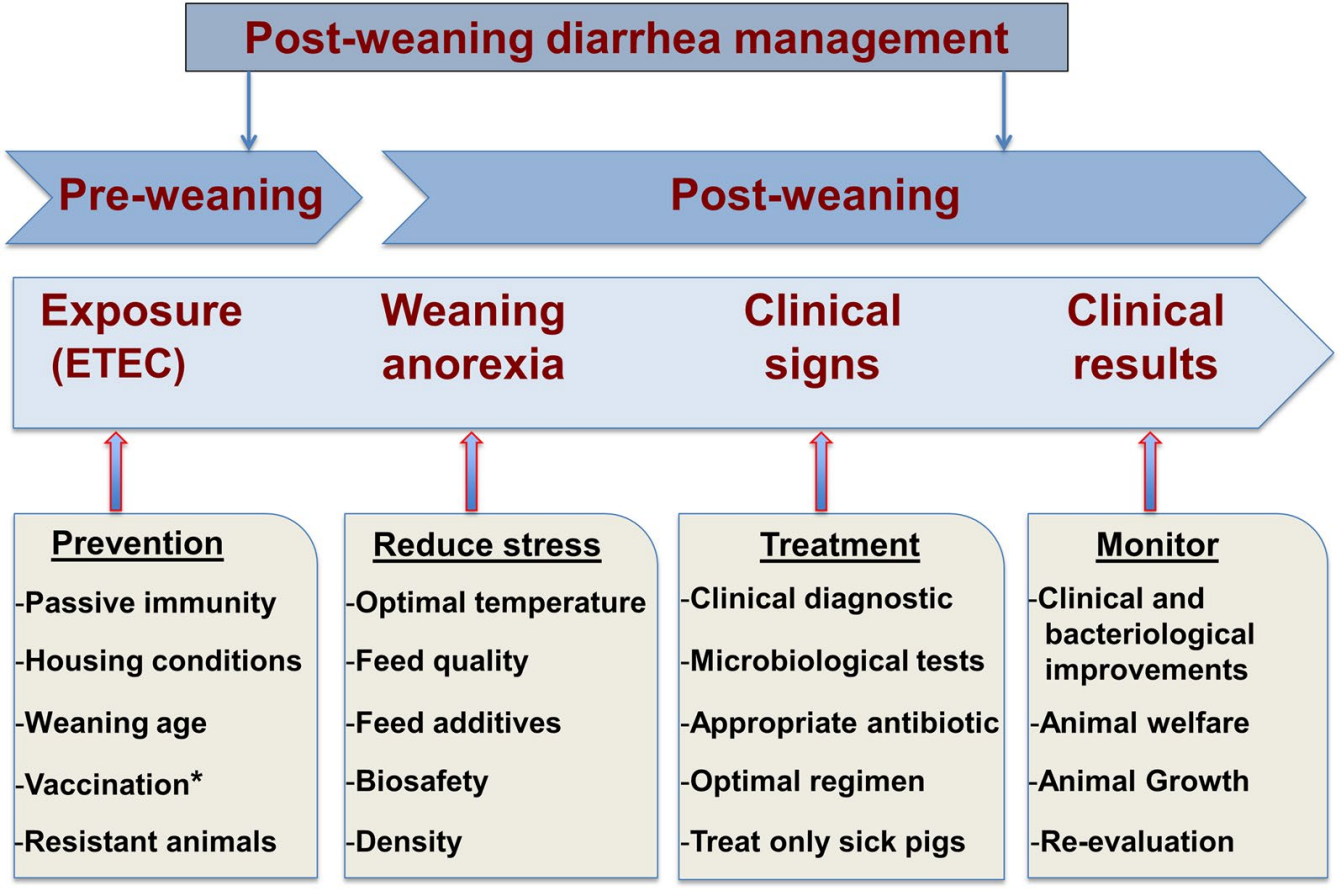
Illustrative interventions for the management of post-weaning diarrhea in pig farms. *Asterisk* Vaccination just prior to or at weaning (Inspired from [159])

For the management of PWD in different stages of its evolution, we propose a comprehensive approach that involves producers, the nutrition industry, veterinarians, the diagnostic laboratory, and researchers (Fig. 3). The absence of a well-identified etiology of PWD and of an effective alternative to antimicrobials requires a close collaboration between the different stakeholders to reduce antibiotic resistance and economic losses caused by this disease in swine.

## Conclusions

Despite the progress that has been observed in modern pig farms during the last decade to prevent infectious diseases and improve global animal health, PWD remains a problem that causes significant economic losses in pig production. Antibiotics have contributed significantly to mitigate the economic losses caused by infectious diseases and particularly PWD in swine. However, increasing bacterial resistance leading to therapeutic failures on farms as well as the greater vigilance of consumers regarding antimicrobial residues, have resulted in more intensive research and a large number of clinical trials for the development of alternatives to antimicrobials. Thus, several alternatives have been developed, some of which have been commercialized for the management of PWD in pigs. However, the effectiveness of these news therapies has been variable from one farm to another due to the management of livestock and farm conditions. Although some alternatives have shown comparable efficacy to antimicrobials or colistin in the control of PWD, there is still a considerable gap between these alternatives and antibiotics concerning their effectiveness in PWD control. Control of housing conditions and vaccination are the most promising strategies for the prevention of PWD in pigs and for reducing of the overall use of antimicrobials on farms. However, the establishment and the effectiveness of these strategies depend on the involvement of all stakeholders in pig farming. Judicious use of antimicrobials in pigs and continued development of alternatives to antimicrobials and colistin remains a priority to ensure a long-term sustainable development in pigs.
